# Antigenic Differences between Normal Hamster Kidney and Stilboestrol Induced Kidney Carcinoma: Complement Fixation Reactions with Cytoplasmic Particles

**DOI:** 10.1038/bjc.1956.65

**Published:** 1956-09

**Authors:** E. Weiler


### ANTIGENIC DIFFERENCES BETWEEN NORMAL HAMSTER KIDNEY AND STILBOESTROL INDUCED KIDNEY CARCINOMA: COMPLEMENT FIXATION REACTIONS WITH CYTOPLASMIC PARTICLES

E. WEILER*

*From the Chester Beatty Research Institute: Institute of Cancer Research, The Royal Cancer Hospital, London, S.W.3*

Received for publication February 28, 1956

It has been shown previously (Weiler, 1952*a*, 1952*b*) that the organ specific antigen of rat liver cytoplasmic particles could not be found in primary hepatoma induced by 4-dimethylaminoazobenzene. Hepatome particles, on the other hand, did contain antigenic properties which were not present in normal liver, which could, however be found in other organs of the rat.

It seemed important to know whether these qualitative antigenic changes during carcinogenesis are a peculiar characteristic of the liver tumour, or whether they are of more general significance in tumour formation.

The stilbestrol induced kidney carcinoma of the golden hamster seemed to provide suitable material for the comparison of malignant tissue with its homologous normal tissue as to their antigenic properties. The tumour is known to arise from the tubular epithelium of the kidney cortex (Horning and Whittick, 1954). On the other hand, organ specific antigenic properties have been shown to be present in the kidney cytoplasmic particles (Henle, Chambers and Groupé, 1941; Furth and Kabat, 1941). It had to be found, therefore, whether the kidney tumour cytoplasmic particles contain kidney specific antigen, and whether in the tumour tissue a new antigen might be present which is not contained in normal kidney.

### MATERIALS AND METHODS

### *Tumours*

The hamster strain of the Chester Beatty Institute was used throughout. Tumours arose in the kidneys 8–12 months after the implantation of stilboestrol pellets, as studied in detail by Horning (1954) and Horning and Whittick (1954). The size of the tumours which were used for the preparation of cytoplasmic particles varied from 5 to 15 mm. in diameter. Only tumour tissue which appeared to be non-necrotic was used.

*Preparation of cell fractions.*—Tumour or whole normal kidneys, freed from their capsule, were used either fresh, directly after dissection from the animal, or after up to several weeks storage at $-35°$ C; in the latter case, tissues had been quickly frozen in test tubes immersed in dry ice-alcohol before being transferred to the deep-freeze cabinet.

The tissues were homogenized in 9 volumes buffered sucrose solution (0·25 M sucrose, M/100 phosphate buffer pH 7·3) by means of a Potter-Elvehjem glass

* Present address: Max-Planck-Institut für Virusforschung, Tübingen, Germany.

homogenizer. The homogenates were layered in centrifuge tubes over a sucrose solution of somewhat higher molarity, and spun at 2000 *g*. for 8 min. The sediment (" nuclear fraction ") was usually resuspended and centrifuged a second time, the second supernatant then being combined with the first. The combined supernatants were centrifuged for 10 min. at 10,000 *g*. in an MSE refrigerated centrifuge, yielding the mitochondrial fraction as sediment. The remaining supernatant was spun either for 30 min. at 60,000 *g*. in a Spinco L centrifuge, or for 90 min. at 20,000 *g*. in the high speed head of the International refrigerated centrifuge. The sediment so obtained was the microsome fraction. Both microsomes and mitochondria were washed once by resuspending and centrifuging again at the same speed. The fractions were suspended in buffered sucrose solution, and either used fresh for serological tests, or stored at — 35° C. after quick-freezing in solid $CO_2$-alcohol mixture. All operations were carried out in the cold.

*Antisera*

Rabbits were injected three times weekly, with increasing amounts of antigen, by the intravenous route. The final two injections were given intravenously and intraperitoneally as well. The injection procedure lasted 3 to 4 weeks, and a total of 3 to 6 mg. antigen nitrogen were given to each rabbit.

Absorption experiments were carried out using cytoplasmic particles from kidney, liver, lung, and sheep red cells as absorbing antigens. Except for kidney, where combined microsome and mitochondrial fractions were used, the particles for absorption experiments were prepared by centrifugation for 80 min. at 20,000 *g*., after previously removing the nuclear fraction at low speed. Thus, the particle preparations represented mixtures of microsomes and mitochondria. In the case of lung, the nuclear fraction was sometimes also used, in addition to cytoplasmic particles, as absorbing antigen.

For the actual absorptions, antiserum and particles were incubated at 37° C. for 30 minutes, and then kept in the refrigerator for 1 or 2 days. Precipitate and particles were removed by centrifugation, and the procedure was repeated, until at least 0·05 ml. gave no complement fixation reaction with the antigen used for absorption ; 4 to 6 absorptions were usually necessary to achieve this, with total amounts of antigen up to 2 mg. antigen N for each ml. of antiserum.

In absorption experiments with sheep cells, about 0·2 ml. of packed cells were added to each ml. of antiserum, kept for 30 min. at 37° C. and then for about 2 hours in the refrigerator. This was repeated once. Thereafter, no Forssman antibody could be detected in the antisera.

*Serological tests.*—The serological procedure has been described elsewhere (Weiler, 1956). In brief, serial twofold dilutions of either antiserum or antigen were carried out. The activity of antisera was estimated by setting up an antiserum dilution series against a constant amount of 8 $\mu$g. N antigen. The activity of a given particle preparation was determined in dilution series of antigen reacting with constant antiserum excess ; the amount of antiserum in these titrations was always five times the minimum amount of serum, which gave at least 3 + reaction in the antiserum dilutions carried out previously. Several checkboard experiments (varying both antiserum and antigen at the same time) have been carried out, using kidney antisera and kidney cytoplasmic particles ; by this it could be secured that the fivefold minimum amount of antiserum, as used in the antigen titrations, really represented antiserum excess.

### RESULTS

### *Antigenic spectrum of cytoplasmic particles from normal kidney*

Unabsorbed antisera against kidney microsomes and kidney mitochondria show complement fixation reactions with cytoplasmic particles prepared from several hamster organs ; moreover, they contain Forssman antibodies, as indicated by sheep red cell haemolysis. Exhaustive absorption with liver particles does not eliminate Forssman antibodies nor antibodies reacting with lung particles. After elimination of Forssman antibodies by a subsequent absorption with red cells, there still remain antibodies reacting with lung particles. If these absorptions are followed by a third absorption with lung particles, the antisera react neither with particles from liver or lung nor with those prepared from spleen, heart, or testes. As these antisera still react with kidney particles, they are regarded as kidney specific.

The relative amounts of antibodies in antisera against kidney cytoplasmic particles can be seen from Table I. Judged by the minimal amounts reacting with

TABLE I.—*Minimum Amounts of Antiserum (ml.) Reacting with 8 μg. N of Homologous Antigen. 2 Units of Complement*

| | Absorbed with | | | | |
|---|---|---|---|---|---|
| | Successively. | | | | Separately. |
| Antiserum against— | — | Liver. | Sheep cells. | Lung. | Kidney. |
| KiC | 0,00 125 | 0,004 | 0,008 | 0,01 | 0,1 |
| | 4 + | 3 + | 3 + | 3 + | 0 |
| KiT | 0,0005 | 0,002 | 0,004 | 0,0025 | 0,1 |
| | 4 + | 4 + | 4 + | 2 + | 0 |
| TuC | 0,001 | 0,001 | 0,025 | 0,07 | 0,01 |
| | 4 + | 3 + | 3 + | 0 | 3 + |
| TuT | 0,0005 | 0,001 | 0,025 | 0,05 | 0,02 |
| | 2 + | 4 + | 3 + | 0 | 3 + |

Degree of reaction with endpoint antiserum volume : 4 + = no haemolysis ; 0 = complete haemolysis. 3 + to 1 + = intermediate values.

Abbreviations : KiC = kidney microsomes. KiT = kidney mitochondria
TuC = Tumour microsomes. TuT = Tumour mitochondria.

8 μg. N of homologous kidney, antigen absorption with liver particles removes about 60 per cent of the antibodies, subsequent absorption with sheep cells another 20 per cent, and finally absorption with lung particles another 10 per cent, thus leaving about 10 per cent kidney specific antibodies These figures concern anti-microsome sera ; anti-mitochondrial sera have about 20 per cent of their activity left as kidney specific antibody after the absorption procedure.

The content in particle fractions of antigens with different specificity can be tested by setting up serial dilutions of antigens against antiserum excess. As can be seen from Table II, kidney microsomes show about half the antigenic activity when reacting with kidney specific anti-microsome serum, as when reacting with unabsorbed serum. Mitochondria retain even more of their activity in reactions with absorbed antisera. These experiments indicate that in microsomes about half of all antigenic groups and in mitochondria even more than half are kidney specific.

There is a pronounced similarity between microsomes and mitochondria as to their reactions with homologous absorbed or unabsorbed antisera. It should be possible to detect differences between both types of particles by cross reactions with the respective heterologous antisera. As can be seen from Table II the reactions of microsomes with anti-mitochondrial sera, or of mitochondria with anti-microsomal sera, are very slightly weaker than the reactions of both types of particles with their homologous antisera. This difference, however slight, was reproducible and somewhat more pronounced in checkboard tests, varying the amounts of both antiserum and antigen in one experiment. From the slight quantitative difference between microsomes and mitochondria, as indicated in these experiments, it can be concluded (as far as the reactions with kidney specific antisera are concerned) that the kidney specific antigen is not quite uniform.

TABLE II.—*Minimum amounts of antigens (µg. N) reacting with various antisera. Antiserum excess*

| Antiserum against. | Absorbed with— | Antigen. KiC. | KiT. | TuC. | TuT. |
|---|---|---|---|---|---|
| KiC | — | 1 (4 +) | 1 (3 +) | 1 (3 +) | 1 (2 +) |
| | Liver | 2 (4 +) | 2 (3 +) | 2 (4 +) | 2 (3 +) |
| | Sheep cells | 2 (3 +) | 1 (3 +) | 8 (3 +) | 8 (4 +) |
| | Lung | 2 (4 +) | 1 (2 +) | — | — |
| KiT | — | 1 (3 +) | 1 (4 +) | 1 (3 +) | 1 (3 +) |
| | Liver | 2 (4 +) | 1 (3 +) | 2 (3 +) | 2 (3 +) |
| | Sheep cells | 2 (3 +) | 1 (3 +) | 4 (2 +) | 8 (4 +) |
| | Lung | 2 (3 +) | 1 (3 +) | — | — |
| TuC | — | 2 (4 +) | 2 (3 +) | 0,5 (3 +) | 1 (3 +) |
| | Liver | 2 (2 +) | 4 (3 +) | 2 (4 +) | 2 (3 +) |
| | Sheep cells | 2 (2 +) | 4 (3 +) | 4 (4 +) | 4 (2 +) |
| | Lung | — | — | — | — |
| | Kidney | — | — | 4 (3 +) | 4 (3 +) |
| TuT | — | 4 (4 +) | 4 (3 +) | 0,5 (2 +) | 1 (2 +) |
| | Liver | 8 (4 +) | 8 (4 +) | 2 (4 +) | 2 (3 +) |
| | Sheep cells | 8 (4 +) | 8 (4 +) | 4 (4 +) | 8 (4 +) |
| | Lung | — | — | — | — |
| | Kidney | — | — | 4 (3 +) | 8 (4 +) |

Sera were absorbed with liver, sheep cells and lung successively, with kidney separately. Degree of reaction with endpoint antigen amounts (in brackets). Compare legend of Table I.— =no detectable reaction.

### *Antigenic spectrum of cytoplasmic particles from kidney carcinoma*

Antisera against tumour particles react with particles from other organs as well. A small part of tumour antibodies can be absorbed with liver particles, leaving a strong Forssman antibody activity in the sera. By subsequent absorption with sheep red cells, a great deal of the over-all activity is removed ; only 2 or 4 per cent respectively of the antibodies reacting with homologous antigen remain in the tumour antisera after successive absorptions with liver particles and sheep red cells (Table I). If the antisera—in a third absorption step—are absorbed with lung particles, no significant reaction with homologous antigen remains detectable. This means that the tumour antisera do not contain an antibody corresponding to the kidney specific antibody in kidney antisera ; for in the latter case the kidney specific antibodies could not be absorbed with particles from liver, lung, and sheep red cells.

In tests with constant a mount of antiserum, serial dilutions of both tumours microsomes and mitochondria give strong reactions with unabsorbed homologous antisera. The reactions with tumour antisera absorbed with liver particles alone or with liver particles and sheep red cells are not uniform. when different tumour preparations are employed. Whereas with unabsorbed tumour antisera microsomes and mitochondria give strong reactions in 0·5 μg. or 1 μg. quantities, the minimum antigenic dose for 3 + reaction varies between 2 μg. N and 8 μg. N, when the particles are tested against tumour antisera absorbed with liver or liver and sheep cells (Table II).

*Cross reactions between kidney and tumour particles*

(a) *Kidney antisera.*—With unabsorbed kidney antisera, tumour particles give a somewhat weaker reaction than with their homologous antisera. The reaction grows progressively weaker, when antisera successively absorbed with liver particles and with sheep red cells are employed in the test. With kidney specific antisera, i.e. sera absorbed successively with liver, red cells, and lung particles, neither tumour microsomes nor tumour mitochondrria give any reaction, employing antigen doses up to 32 μg. N. That is, the kidney specific antigen could not be found in tumour particles. The tumour nuclear fraction or the soluble supernatant remaining after the sedimentation of the microsomes also gave no reaction with kidney specific antisera.

This result was obtained with all tumour preparations tested, the major part of which consisted of pooled tumours. Freezing and storing of tumour tissue before fractionation, or freezing and storing of cytoplasmic fractions, had no effect on the result of this experiment, as could be demonstrated by parallel tests employing fresh material and material stored in the frozen state.

(*b*) *Tumour antisera.*—Kidney particles give a considerably weaker reaction with unabsorbed tumour antisera than with their homologous antisera. The reaction grows progressively weaker after absorption of tumour antisera with liver particles or with liver particles and sheep red cells (Table II).

To test the possibility that tumour antisera contain " tumour specific " antibodies, absorption experiments were carried out employing kidney particles as absorbing antigen. After exhaustive absorption of antitumour sera with kidney particles, a small fraction of antibodies reacting with tumour particles remained in the sera. In antimicrosome serum, about 10 per cent of the activity remains after absorption with kidney particles. in antimitochondrial serum only about 2·5 per cent (Table I). The experiment was repeated three times, employing different tumour preparations as test antigens, with consistent results.

Tumour antigen titrations, employing constant amounts of kidney absorbed antitumour sera, reveal that the minimum amount of antigen necessary for 3 + reaction was 4 or 8 μg. N. Compared with reactions employing unabsorbed antisera, the " tumour specific " antigenic activity of tumour particles is very weak (Table II).

From these experiments it can be concluded that in tumour cytoplasmic particles there is an antigenic component which is not present in kidney cells. This is, however, not " tumour specific " as all antibodies from tumour antisera can be eliminated by successive absorptions with heterologous antigens : liver, lung, and sheep red cells.

In all experiments with tumour cytoplasmic particles, microsomes, and mitochondria resembled each other qualitatively, and showed very similar quantitative reactions (Table II).

### DISCUSSION

Cytoplasmic particles from kidney carcinoma appear not to contain the organ specific antigen which is present in the microsomes and mitochondria from normal kidney. This result was obtained by complement fixation tests in which tumour particles gave no reaction with organ specific antisera against particles from normal kidney.

Quantitatively, one μg. N of normal particles gave a 4 + reaction, whereas no significant reaction could be obtained with tumour particles, using up to 32 μg. N of antigen. Assumming a quantitative difference rather than a qualitative one, the kidney specific antigen content of tumour cytoplasmic fractions would be at least 64 times lower than that of normal particles. In the latter the specific antigen constitutes about half of the total serological activity.

The most striking result is the complete resemblance in the serological characters between the kidney carcinoma and the butter-yellow induced rat hepatoma (Weiler, 1952*a*, 1952*b*), each compared with their respective homologous organ. In both cases the organ specific antigen of the cytoplasmic particles is lost.

It seems reasonable to postulate, therefore, that in normal cells organ specific antigens are a chemical expression of the morphological and functional differentiation, and part of the organ specific growth regulating system. Their loss during carcinogenesis leads to dedifferentiation and unlimited growth. The " deletion hypothesis " has been discussed in more detail by Miller and Miller (1953), Druckrey (1954), Rusch (1954), and more recently, by Haddow (1955). In future work the chemical nature and physiological significance of organ specific antigens will have to be investigated.

In yet another respect there is a resemblance between hepatoma and kidney carcinoma : both tumours contain in their cytoplasmic particles an antigenic component not present in their homologous normal organs. This could be demonstrated by the presence of antibodies in antitumour sera after complete absorption with particles from the normal organ. The antibodies demonstrated in this way are, however, not "tumour specific ", as they could be absorbed by antigens from heterologous organs ; in the case of kidney tumour antisera, the whole serological activity could be eliminated by successive absorptions with liver particles, sheep cells (Forssman antibody), and lung particles. Compared with the liver-hepatoma system, however, the serologic activity of kidney absorbed antisera as well as the activity of tumour particles reacting with excess of this antiserum is very low. This indicates, that the tumour antigens, which are not contained in kidney, but in heterologous organs, play only a minor role in the antigenic spectrum of the kidney carcinoma cell.

### SUMMARY

The antigenic relationships between microsomes and mitochondria from normal hamster kidney and from stilboesterol induced kidney carcinoma have been investigated. Rabbit antisera against cytoplasmic particles from normal and malignant tissue were used. The antigenic activity of cytoplasmic particles was determined in complement fixation tests.

Antisera against kidney cytoplasmic particles were rendered kidney specific by successive absorptions with liver particles, sheep red cells, and lung particles. Tumour microsomes and mitochondria did not react with kidney specific antisera. This indicates that tumour cells do not contain the organ specific antigen of normal kidney cytoplasmic particles.

The whole activity of tumour antisera could be abolished by successive absorptions with liver particles, sheep red cells. and lung particles. That means that tumour antisera do not contain antibodies corresponding to the kidney specific antibodies.

When tumour antisera were exhaustively absorbed with kidney particles, there remained a weak activity towards the homologous antigen ; tumour particles, therefore, seem to contain a small antigenic component which is not present in normal kidney, which can, however, be found in other organs of the hamster.

With unabsorbed antisera, quantitative differences were detectable between cytoplasmic particles from normal and malignant tissue, when cross reactions with the respective heterologous antisera were carried out.

The antigenic differences between normal and malignant tissue, as reported in the present paper, were very similar to those found previously in liver carcinogenesis.

I am much indebted to Professor A. Haddow for providing facilities in the Chester Beatty Institute, for helpful suggestions, and for his interest in the work ; and to Dr. E. S. Horning for valuable discussion, and for providing the kidneys from hamsters treated with stilboestrol in his experiments.

This investigation has been supported by grants to the Chester Beatty Research Institute from the British Empire Cancer Campaign, the Jane Coffin Childs Memorial Fund for Medical Research, the Anna Fuller Fund and the National Cancer Institute of the National Institutes of Health, U.S. Public Health Service.

Thanks are also due to the British Council for granting a British Council Scholarship.

### REFERENCES

DRUCKREY, H.—(1954) in ‘ Zweites Freiburger Symposium : Grundlagen und Praxis chemischer Tumorbehandlung,’ 1–27. Springer-Verlag.

FURTH, J. AND KABAT, E. A.—(1941) *J. exp. Med.*, **74**, 257.

HADDOW, A.—(1955) *Ann. Rev. Biochem.*, **24**, 689.

HENLE, W. CHAMBERS, L. A. AND GROUPÉ, V.—(1941) *J. exp. Med.*, **74**, 495.

HORNING, E. S.—(1954) *Brit. J. Cancer*, **8**, 627.

*Idem* AND WHITTICK, J. W.—(1954) *Brit. J. Cancer*, **8**, 451.

MILLER, J. A. AND MILLER, E. C.—(1953) *Advanc. Cancer Res.*, **1**, 339.

RUSCH, H. P.—(1954) *Cancer Res.*, **14**, 407.

WEILER, E.—(1952*a*) *Z. Naturf.*, **7b**, 327. (1952*b*) Thesis, Tubingen. (1956) *Z. Naturf.*, **11b**, 31.


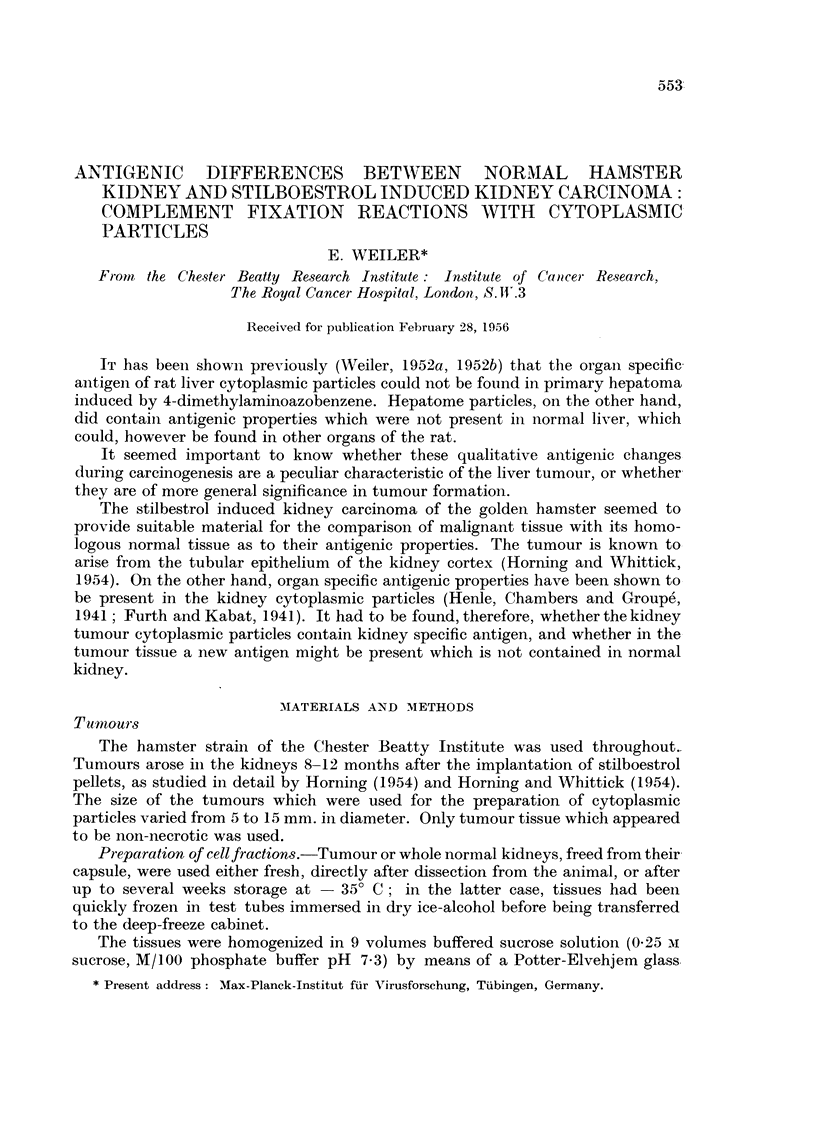

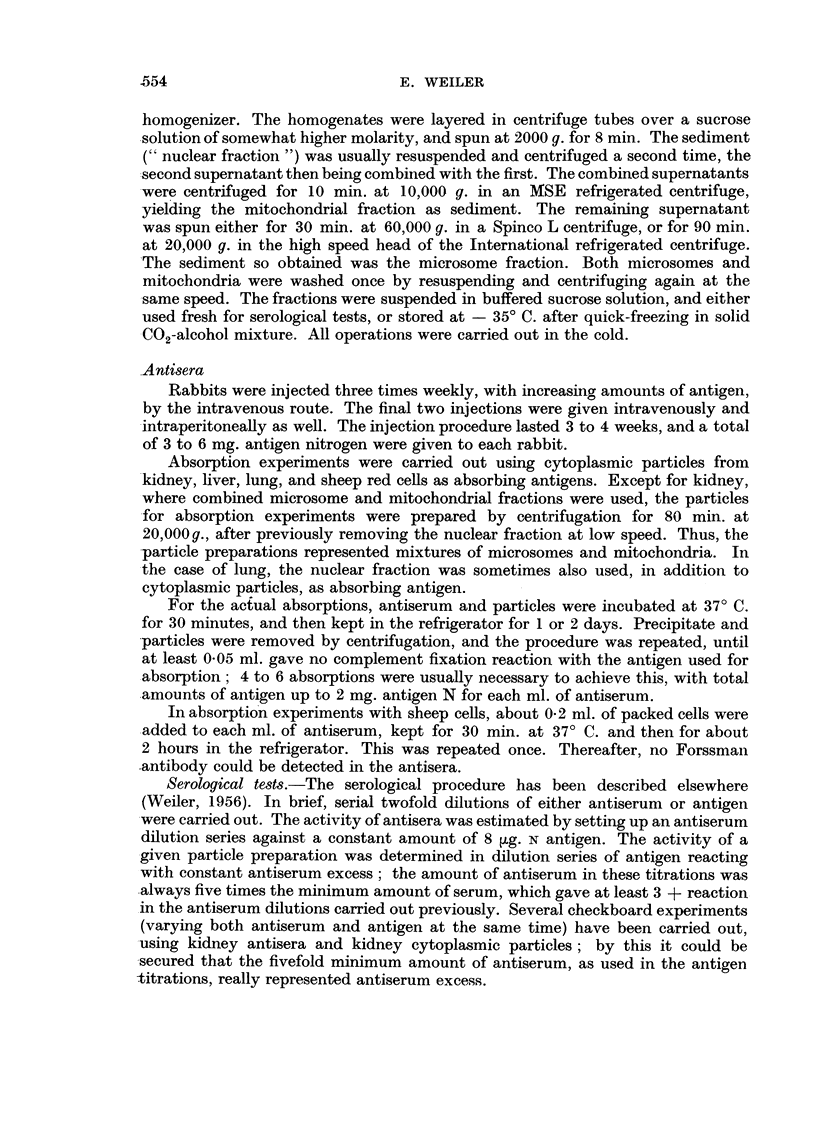

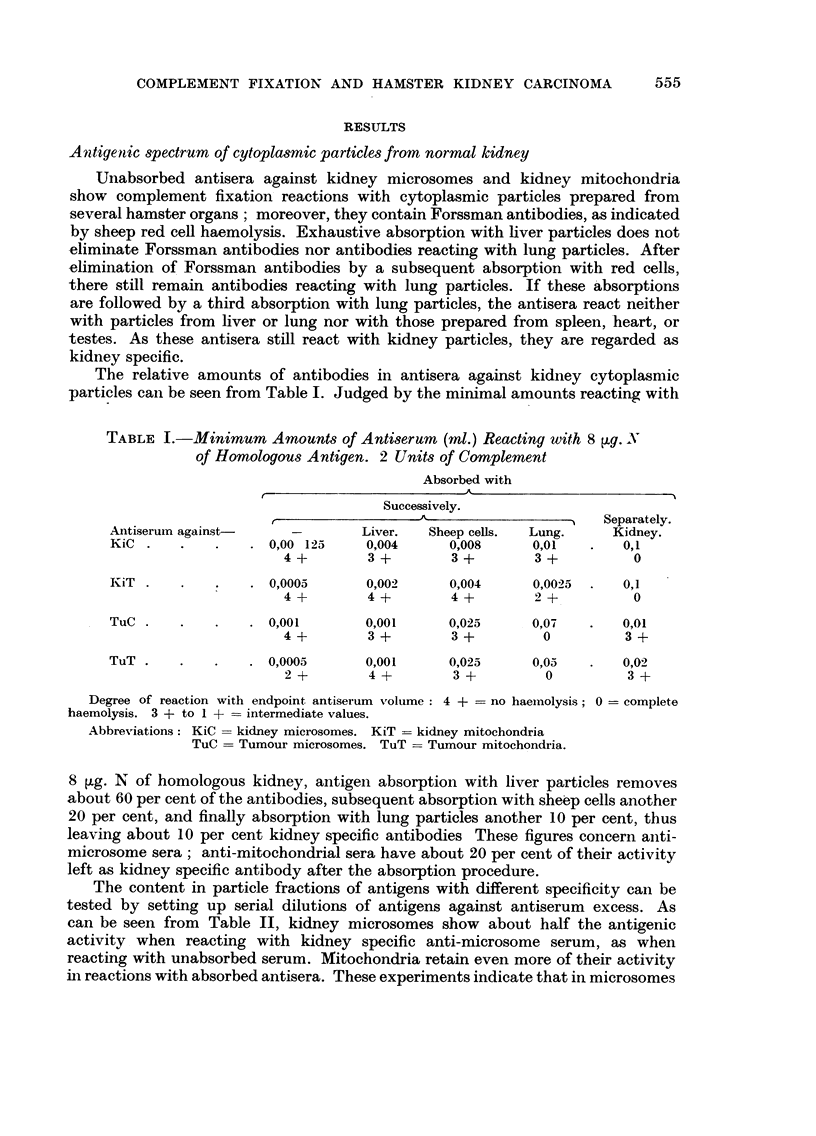

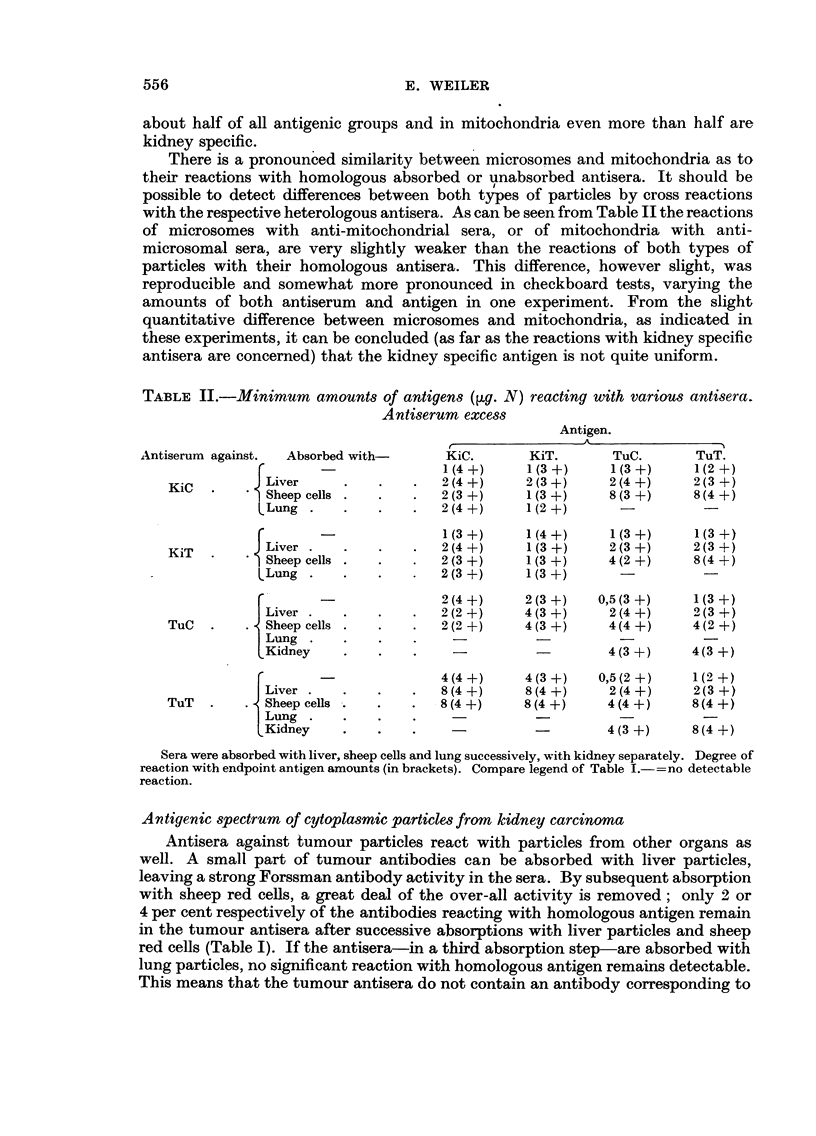

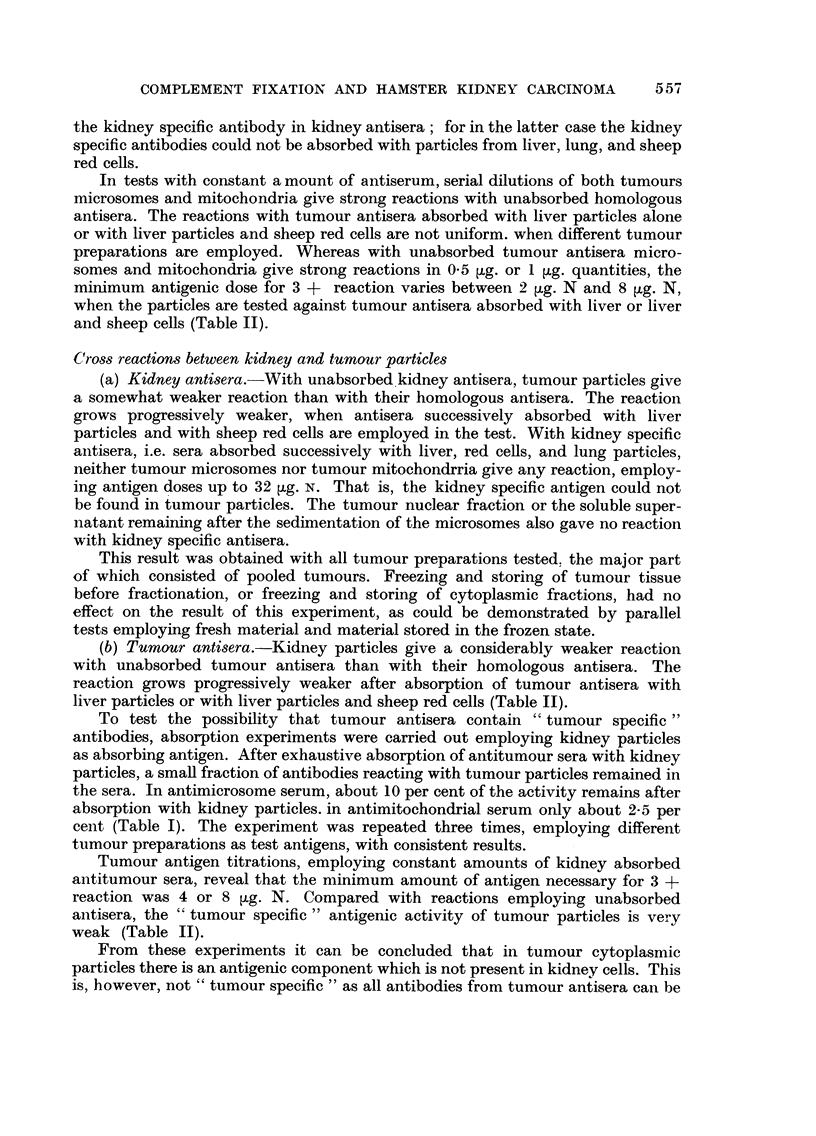

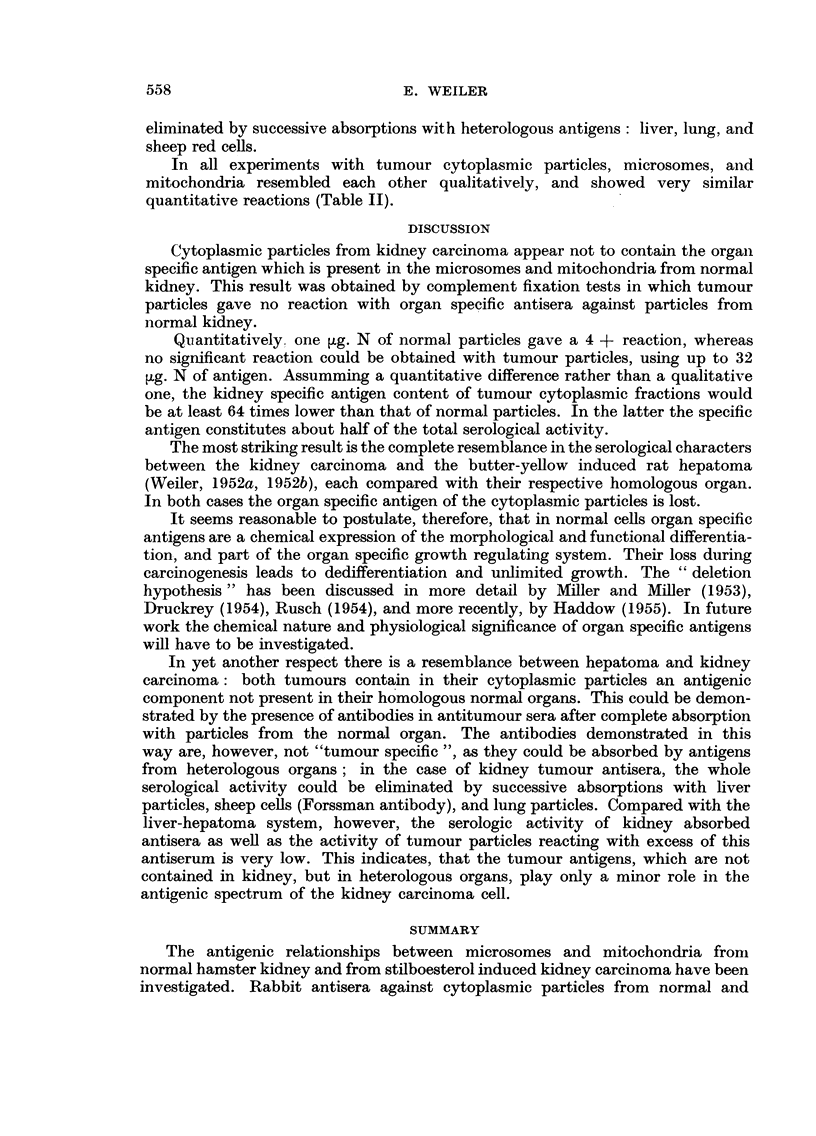

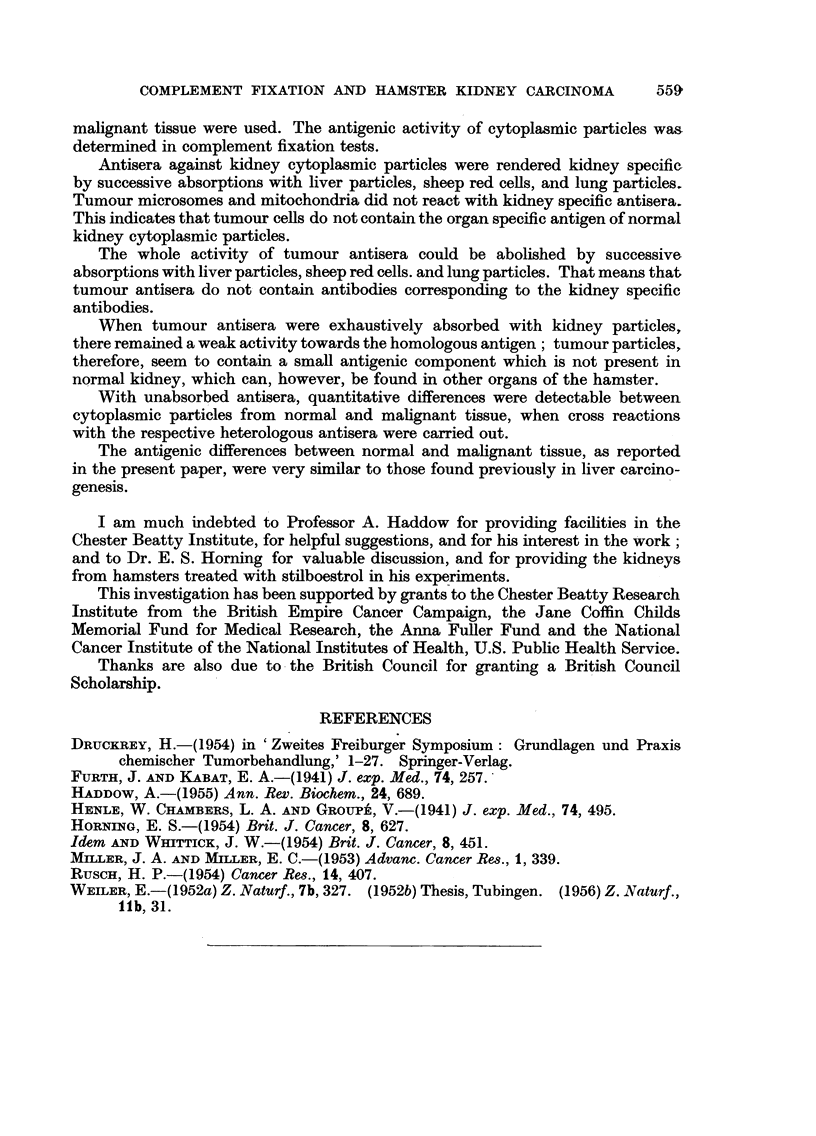


## References

[OCR_00430] HADDOW A. (1955). The biochemistry of cancer.. Annu Rev Biochem.

[OCR_00435] HORNING E. S., WHITTICK J. W. (1954). The histogenesis of stilboestrol-induced renal tumours in the male golden hamster.. Br J Cancer.

[OCR_00438] RUSCH H. P. (1954). Carcinogenesis; a facet of living processes.. Cancer Res.

